# Sex and autoimmune disease: Four mechanisms pointing at women

**DOI:** 10.31138/mjr.30.3.162

**Published:** 2019-09-30

**Authors:** Angela Ceribelli, Maria De Santis, Carlo Selmi

**Affiliations:** 1 Rheumatology and Clinical Immunology, Humanitas Clinical and Research Center, IRCCS, Rozzano, Italy,; 2 BIOMETRA Department, University of Milan, Italy

**Keywords:** autoimmunity, autoantibody, twins, microbiome, sex chromosome, estradiol

## Abstract

The ultimate goal of modern medicine is a personalized approach being tailored on the single patient, ie, tailored, based on a finely tuned definition of the immunogenetics, epigenetics, microbiome, and biomarkers, to maximize results and minimize risks particularly of new targeted treatments. Among individual factors around which to tailor the patient management are sex and age, with gender-medicine finally becoming central to the research agenda. Of note, we are not convinced that a whole personalized medicine approach in its current form will necessarily include gender medicine and thus this should remain central to the research agenda. To tackle this crucial issue, however, we should first be able to answer a question of paramount importance, that is, why does autoimmunity affect women more than men? The growing number of experimental works in this area militate against an easy answer to this question, but we will herein briefly discuss four major candidates (sex hormones, sex chromosomes, environmental factors, and the microbiome) to which some unsuspected others may be ancillary.

## INTRODUCTION

The link between autoimmunity and specific predisposing factors to rheumatic diseases is still unknown, but it is clear in particular from twin studies that genetic predisposition is not enough, and that epigenetic mechanisms are fundamental to trigger the rheumatic disease clinical onset.^1,2^ In particular, environmental factors can modify mechanisms such as DNA methylation that influence gene expression without changing the DNA sequence, and in recent years, the role of microbiota is considered another significant aspect in rheumatic disease pathogenesis. In the present manuscript we will discuss these points with a particular focus on the link between epigenetics and rheumatic diseases in female patients, with the last findings on epigenetics of sex chromosomes in particular.

## SEX HORMONES AND AUTOIMMUNITY: WHY WOMEN?

According to the last statistics of the World Health Organization (WHO) published in 2019 (https://www.who.int/gho/publications/world_health_statistics/2019/en/), women outlive men in particular in wealthy countries. About 33/40 of the leading causes of death contribute to reduced life expectancy in men more than in women, and even if they face the same disease, men often seek healthcare less than women. Moreover, men are much more likely to die from preventable and treatable non-communicable diseases and because of road traffic accidents. In 2016, the probability of a 30-year-old dying from a non-communicable disease before 70 years of age was 44% higher in men than in women.

Differences between male and female are clear also in their susceptibility to autoimmune diseases but also to infectious diseases and cancer types.^3^ In fact, females show susceptibility to systemic and localized autoimmune diseases such as Rheumatoid Arthritis (RA), Systemic Lupus Erythematosus (SLE), Hashimoto Thyroiditis and Type 1 diabetes, while male patients are more prone to develop non-reproductive cancers (bladder, bowel, kidney, leukemia, malignant melanoma) and specific infectious diseases such as Hepatitis B and tuberculosis.^3^ These infections are more associated to pathogen damage in males, while in females, they mostly depend on the reproductive status, including pregnancy, as in the case of toxoplasmosis.^3^

Female individuals show a more active immune response compared to males, with significant differences in both innate and adaptive immunity compared to male subjects.^3^ In particular, the innate immune response in females is strongly dependent on TLR7 expression, APC efficiency, type I interferon activity, phagocytic capacity of macrophages and increased IL-10 production.^3^ On the contrary, male subjects have stronger production of IL-10 mediated by TLR9 and TLR4, higher number of NK cells and increased capacity to produce pro-inflammatory cytokines.^3^ As for sex differences in the adaptive immune system, females have increased B cell number and antibody production, and higher CD4+ T cell count and CD4/CD8 T cell ratio compared to males, with stronger T cell proliferation.^3^

In the life of female patients, pregnancy is a moment with a strong impact on the immune system, in particular in female patients suffering of RA and SLE. In healthy females, during pregnancy the maternal immune system is activated by the action of pathogens, allergens and self-antigens, and also the fetoplacental tissues and pregnancy-associated hormones are very important in maintaining tolerance towards the fetus.^4,5^ Pregnancy in SLE patients is associated to the increased production of anti-inflammatory factors such as IL-4, IL-10, TGF-β, M2 macrophages, Th2 cells, regulatory T cells and antibody production.^6,7^ On the contrary, RA patients during pregnancy have a reduction in proinflammatory factors such as IL-12, IL-2, IFN-γ, TNF-α, M1 macrophages, Th1 and Th17 cells.^8,9^

The differences in the immune response between male and female subjects also depend on the age period we are considering. In fact, for aspects such as the pro-inflammatory response, differences are strong during puberty while they tend to disappear later, probably because of hormonal effects. For other aspects there is no specific difference from birth to old age, as for example in the adaptive immune response which shows higher CD4+ T cells numbers and CD4/CD8 ratio in females.^3^ It is known that SLE onset is mainly in young female patients during child-bearing age, but hormonal changes during menopause may have an impact on SLE disease course as demonstrated.^10^ Although premenopausal SLE patients have more active disease compared to postmenopausal women with SLE, the authors demonstrate a constant rate of improvement over time, and this does not seem to be related to change according to the menopausal status. Authors conclude by saying that clinicians should not anticipate the postmenopausal era in a SLE patient’s course as a period of natural disease improvement.^10–12^

## SEX CHROMOSOMES AND TWINS: WHAT IS THE ROLE OF GENETICS IN AUTOIMMUNE DISEASES?

When considering the role of X chromosomes in the susceptibility to autoimmune diseases, it is known that a significant number of sex and immune-related genes are present in the X chromosome.^13,14^ Moreover, specific immunodeficiencies are secondary to X-linked mutations and X chromosome disorders such as Turner’s Syndrome are associated with increased risk of autoimmune diseases.^15–18^ In the case of primary biliary cholangitis (PBC), the rate of X monosomy is significantly higher than in controls and it increases with age, thus haploinsufficiency for specific X-linked genes may increase female susceptibility to PBC.^19,20^ The role of X monosomy has been demonstrated also in systemic autoimmune rheumatic diseases such as Systemic Sclerosis (SSc), in which X monosomy rate is more frequent in peripheral T and B lymphocytes than in other blood cells, and there is no male fetal microchimerism.^21,22^ The genetic mechanisms at the basis of the onset of autoimmune diseases have been extensively performed also in populations of monozygotic (MZ) twins, because they share an identical genetic background and high concordance rates between MZ twins suggest a genetic predisposition, while low concordance supports the role of environmental factors on disease onset.^2,23^ The concordance rates of autoimmune diseases are variable based on the weight of genetic predisposition on disease onset, and it could vary from only 4% in SSc MZ twins to 75–83% for MZ twins affected by celiac disease,^2,24^ while this rate becomes very low up to 0% in case of dizygotic twins. These results clearly support the finding that genes are not fundamental for the onset of autoimmune diseases, and that non-heritable influence dominates the activity of the human immune system^25^ represented by cellular populations activity and expression, cytokines production and response, and production of serum proteins. Thus, MZ twins acquire differences while aging, thanks to a specific “epigenetic drift” which is influenced by environment, and for this reason it is different from one individual to the other, despite the genetic identity in MZ twins. This aspect has been demonstrated in several reports, as by Fraga et al. who showed the presence of differences in methylation activity and histone deacetylation in older MZ twins, affecting the gene expression of the same genotype shared by MZ twins.^26^

**Table 1.** The main risk factors involved in the onset of rheumatic diseases in female patients are arbitrarily subdivided into immune-related, sex-related, environmental, and biological.

Immunological factors	Sexual factors	Environmental factors	Biological factors
Increased type 1 IFN activity and macrophage activity	X chromosome abnormalities	Chemicals such as nail polish and hair dye	Viral infections
Increased B cell number and autoantibody production	Fertile age	UV light exposure	Bacterial infections (eg, periodontal disease)
Increased T cell proliferation	Pregnancy	Smoking and alcohol	Microbiome alterations

As described previously, the role of X chromosome is fundamental in autoimmune diseases, and differentially methylated X-chromosome genes in MZ twins with SSc may be candidate for SSc susceptibility.^22^ This is the case of genes that include transcription factors and surface antigens which may influence pathways involved in apoptosis, cell proliferation, inflammation, oxidative stress in SSc patients.^22^ Similar mechanisms have been studied also in MZ twins discordant for PBC, in which analysis by genome-wide DNA methylation, copy number variation and gene expression show several genes differentially expressed in discordant PBC siblings, thus highlighting the importance of epigenetic difference between the twins.^27^

## WHAT ABOUT THE ROLE OF ENVIRONMENT IN THE ONSET OF AUTOIMMUNE DISEASES?

The role of environment in the onset of autoimmune diseases is controversial but it is part of the multifactorial aspects that are able to trigger autoimmune conditions. Environmental links to autoimmunity have been described, and they range from anecdotal associations or case series to largely investigated experimental and epidemiological studies.^24,28^ This is the case of a condition such as Primary Biliary Cholangitis (PBC),^29^ as described in 2005 by a controlled interview-based study that enrolled 1032 PBC patients. These subjects were administered a modified version of the US National Health and Nutrition Examination Study (NHANES III) questionnaire by trained personnel to evaluate associations between PBC and several other factors including environmental factors such as lifestyle and reproductive factors. The results of this study show that known environmental risk factors for the onset of PBC may be urinary tract infections, chemicals contained in cigarettes, nail polish, and more weakly, the use of hair dye, thus suggesting how many factors together may induce PBC in female patients beside their familial and genetic predisposition.^29^ In the case of systemic autoimmune rheumatic diseases, environmental triggers may be divided in biological, chemical and physical factors, such as viral and bacterial infections, ultraviolet radiation and exposure to agents such as smoke, alcohol and silica.^30^ For example, it has been reported in recent years that exposure to periodontal bacteria such as *Porphyromonas Gingivalis* is able to induce arthritis in animal models of collagen-induced arthritis,^31^ and it is well known that acute and chronic viral infections may trigger an autoimmune response and induce the onset of rheumatic diseases such as HCV and cryoglobulinemia in vasculitis patients.^32^ As for physical factors influencing the onset of rheumatic diseases, UV light is known to be a trigger factor for conditions such as dermatomyositis and SLE, thus leading to the exacerbation of these diseases in particular periods of the year and in different geographical areas depending to UV intensity.^33,34^

## UNSUSPECTED FACTORS IN THE ONSET OF AUTOIMMUNE DISEASES: THE ROLE OF GUT MICROBIOTA

In the last years, we have faced an increased interest on the role of an additional environmental factor related to rheumatic diseases, represented by gut microbiota, as it seems to be involved in the etiology and pathogenesis of several conditions including rheumatic diseases.^35–37^ This has been demonstrated both in animal models and in observational studies involving patients, showing that gut microbiota can influence the immune response and the onset of rheumatic diseases,^36,38^ and alterations in gut microbiota in early life can suppress autoimmunity in animal models at high genetic risk for the disease.^39^ Additional data on human subjects have shown that puberty and pregnancy can modify the intestinal microbiota, causing metabolic changes that may influence not only the onset of an autoimmune disease, but also fertility and reproduction which is a significant issue in young rheumatic patients.^40,41^ Additional data on the role of testosterone in the onset of autoimmune diseases have shown that microbioma alterations that occur in young and commensally colonized mice, they can induce testosterone production and metabolic changes, and they are able to oppose autoimmunity, thus, maintaining fertility.^42^ The female predisposition that we observe in RA declines with advancing of age, and it develops in parallel with a reduction in testosterone,^43^ while this is not observed in other conditions such as Type 1 diabetes, which is not sex-biased, perhaps, because this condition has a peak of onset before puberty, in young children.^39,44^

## CONCLUSIONS

As discussed in the present manuscript, we can conclude that the paradigm of genes versus environment in auto-immune diseases remains unknown for some parts, as general immunity appears to have weak hereditary basis but strong sex skewage. This has been demonstrated in twin studies that represent natural models to determine the role of genetics and epigenetics in autoimmune diseases. A specific interest has grown in the last decades in particular on the study of epigenetics in autoimmunity, as it is the ideal link between genetics and environmental factors. In fact, generating epigenome-wide association studies (EWAS) and GWAS with methylome and chromatin epigenomics will maximize our understanding of complex mechanisms in autoimmunity and rheumatic diseases, but also, the study of gut microbiome points to sex as a major determinant of autoimmunity.
